# Altered expression of T cell Immunoglobulin-Mucin (Tim) molecules in peripheral blood mononuclear cells in aplastic anemia

**DOI:** 10.1186/s12935-014-0144-2

**Published:** 2014-12-17

**Authors:** Xin Liu, Xin Cui, Dai Yuan, Ying Li, Ning-ning Shan, Xin Wang, Yu Hu

**Affiliations:** Department of Hematology, Shandong Provincial Hospital Affiliated to Shandong University, 324 Jing Wu Road, Jinan, Shandong 250021 China; Department of Internal Medicine, Shandong Provincial Hospital Affiliated to Shandong University, Jinan, China; Haematology Oncology Centre, Qilu Hospital, Shandong University, Jinan, China

**Keywords:** Aplastic anemia, T-cell immunoglobulin and mucin domain, IFN-γ, Interleukin 4, Cytokines, RT-PCR

## Abstract

To evaluate the balance between T-cell immunoglobulin and mucin domain (Tim) molecules(Tim)-1 and Tim-3 in patients with aplastic anemia (AA), plasma IFN-γ and IL-4 levels were measured in patients with active AA (n = 41), AA in remission (n = 29) and in healthy subjects (n = 40) by enzyme linked immunosorbent assay (ELISA). Using real-time quantitative polymerase chain reaction (RT-PCR), the mRNA expression of IFN-γ, IL-4, Tim-1 and Tim-3 were studied in all subjects. The results showed that the expression of Tim-3 in newly diagnosed patients was significantly deceased, compared with the controls. Meanwhile, Tim-1 mRNA expression in the active AA group was not significantly reduced, which resulted in a declined ratio of Tim-3/Tim-1 in patients with active disease. During the remission stages, the levels of these transcription factors were comparable with those observed in the healthy controls. These findings are the first data on the expression of the Tim-1 and Tim-3 molecules in AA. The reduced levels of Tim-3/Tim-1 in PBMCs during the active stages of disease suggest that they may play a possible role in the pathogenesis and course of AA.

## Introduction

Acquired aplastic anemia (AA) is mostly considered as an immune-mediated bone marrow failure syndrome, which differs from the other conditions associated with inherited mechanisms [1]. Environmental exposure to, for example, drugs, viruses, chemical and physical toxins, is thought to trigger the aberrant immune response in some patients, but most cases, the disease is classified as idiopathic immune-mediated AA. Aberrant immunity, including abnormal immune cells and molecules, contribute to the development of acquired AA [2,3]. It has become evident that T helper 1 (Th1) and Th2 cells have pathogenetic importance in AA. Specifically, activated antigen-presenting cells (APCs), especially dendritic cells (DCs), may promote the polarization to Th1 cells and activate cytotoxic T lymphocytes (CTLs). A variety of immune molecules, including interferon-γ (IFN-γ), tumor necrosis factor-α (TNF-α) and interleukins (ILs), which are produced by DCs, T lymphocytes and stromal cells, comprise a cytokine network that may contribute to the destruction of hematopoietic stem cells [3-6].

Recent investigations into the mechanisms that regulate the activation and function of CD4+ T cells have shown that T cell immunoglobulin-mucin (Tim) proteins are important regulators of immune function. Tim family members are type I transmembrane proteins, with extracellular immunoglobulin and mucin domains and intracellular domains of different lengths. Tims are differentially expressed on Th1 and Th2 cells [7,8]. The Tim gene family includes eight genes in mice and three genes in human. Studies in mice have indicated that Tim-1 is involved in T helper cell differentiation and suggested that the protein is a positive regulator of T cell activity [9,10]. In humans, the gene encoding the Tim-1 protein has been considered as an important atopy susceptibility gene and is associated with Th2 T cell responses [11], suggesting that Tim-1 controls critical regulatory pathways in the immune system.

Another member of Tim family proteins is Tim-3, which in contrast to Tim-1, preferentially is expressed on fully differentiated CD4+ Th1 cells, but not on Th2 cells, and functions to inhibit aggressive Th1-mediated auto- and allo-immune responses [12,13]. Currently, no studies regarding the expression of Tim-1 and Tim-3 in patients with acquired AA have been reported.

In the present study, we hypothesized that the imbalance of Tim-1 and Tim-3 may play an important role in acquired AA. The mRNA expression levels of Tim-1, Tim-3 and other cytokines were measured in the peripheral blood mononuclear cells (PBMCs) of 41 newly diagnosed patients with active AA, 29 AA patients in remission and 40 healthy volunteers to investigate the possible role that Tim-1 and Tim-3 may play in AA.

## Methods

### Patients and controls

In total, 41 newly diagnosed AA patients (22 females and 19 males; age range, 13–65 years; median age, 28 years) were enrolled in this study. None of them had been transfused or treated by immunosuppressive therapy prior to sampling. Out of the 41 patients, 20 patients were diagnosed with severe AA (SAA) and 21 patients with moderate AA (MAA) according to the criteria of Camitta et al. [14]. The patients’ bone marrow cytogenetics was normal in all cases, and Fanconi anemia was excluded in children and adolescents based on family history and the presence of typical physical characteristics. Patients complicated with diabetes, hypertension, cardiovascular diseases, pregnancy, active infection, or connective tissue diseases, such as systemic lupus erythematosus, were excluded. A total of 29 AA patients (15 females and 14 males, age range 15–54 years, median 38 years) were in remission. All of the patients met the diagnostic criteria of AA by bone marrow biopsy and peripheral blood counts, as recommended by the International Study of Aplastic Anemia and Agranulocytosis [15]. Additionally, 40 healthy controls were included (18 females and 22 males; age range, 12–63 years; median, 34 years). Enrollment took place between July 2011 and January 2014 in the Shandong Provincial Hospital Affiliated to Shandong University. Our research was approved by the Medical Ethical Committee of Shandong Provincial Hospital Affiliated to Shandong University. An informed consent document was obtained from each participant.

### Isolation of RNA from PBMC

Fifteen milliliters of heparinized, venous peripheral blood were collected from all patients and control individuals. The blood was centrifuged and the heparinized plasma was stored at −80°C. Mononuclear cells were isolated from the heparinized blood by gradient centrifugation on Ficoll-Paque (Pharmacia Diagnostics, Uppsala, Sweden). The PBMC were then applied to an RNeasy mini-column (Qiagen GmbH, Hilden, Germany), which was processed according to the manufacturer’s recommendations. Total RNA was eluted with 15 μl of RNase-free water and stored at −80°C. The amount of RNA was determined using the Eppendorf Biophotometer (Brinkmann Instruments, Westbury, NY, USA) and normalized to 1 μg/ml for each subsequent real-time quantitative polymerase chain reaction (RT-PCR) process.

### Enzyme-linked immunosorbent assay (ELISA)

No significant cross-reactivity with human plasma IFN-γ and IL-4 was determined using a commercial ELISA (Jingmei, Beijing, China) according to the manufacturer’s instructions. The lower detection limit of this assay was 2 pg/ml. The samples were run in duplicate.

### Quantitative real-time polymerase chain reaction analysis

Multiplex real-time PCR was performed for IFN-γ, Tim-1, Tim-3 and the endogenous control (β-actin) on ABI PRISM®7500 Sequence Detection System (Applied Biosystems Foster City, CA, USA) using SYBRw Green (Toyobo, Japan) as a double-strand DNA-specific binding dye. Amplification was carried out in a total volume of 20 μl containing 0.5 mm of each primer, 10X SYBRw Green and 0.5 mm of cDNA. The primers for all mRNA assays were intron-spanning. The sequences of the amplification primers are listed in Table 1.

**Table 1:** **Cytokine levels in AA patients and controls (mean ± SD)**

***Groups***	***Numbers of cases***	***IFN-γ(pg/ml)***
Controls	40	11.2 ± 5.2
AA (active)	41	44.2 ± 8.6^*#^
SAA	20	48.8 ± 6.8^&^
MAA	21	39.1 ± 7.5
AA (remission)	29	11.4 ± 4.9

*P < 0.05, AA (active) compared with normal controls;

^#^ *P* < 0.05, AA (active) compared with AA(remission);

^&^ *P* < 0.05, SAA compared with MAA.

The PCR reactions were cycled 40 times after initial denaturation (95°C, 5 min) with the following parameters: denaturation 95°C, 15 s; annealing 60°C, 15 s; extension 72°C, 35 s, with temperature transition rates of 20°C/s. Fluorescence was acquired at extension 72°C, which was below the product melting temperature (Tm), with a hold of 5 s. Melting curve analysis of the amplification products was performed at the end of each PCR reaction. All reactions were carried out in triplicate. ABI Sequence Detection System software version 1.0 (PE Applied Biosystems, UK) was used to determine the cycle number at which the fluorescence emission crossed the automatically determined Ct value. All PCR products were visualized by electrophoresis of 2% agarose gels stained with ethidium bromide.

### Statistical analysis

The plasma levels were expressed as the mean ± SD and statistical significance was determined by one-way ANOVA. All calculations were performed with SPSS (version 16.0; SPSS, Inc., Chicago, IL, USA). We used the comparative Ct method (using arithmetic formulae) for the relative quantification of cytokine mRNA according to relative expression software tool (REST©) [16]. A probability value of *P* < 0.05 was considered statistically significant.

## Results

### Cytokine variations in AA patients

The plasma levels of IFN-γ in AA patients with active disease (*P* < 0.05) and AA patients in remission (*P* < 0.05) were significantly increased compared with normal controls. Furthermore, the expression level of IFN-γ in SAA patients were higher than that in MAA patients (Table 1). No significant differences were found between the patients in remission and the normal controls. The levels of IL-4 were below the detectable limit of the assay used. There was no correlation between the IFN-γ or IL-4 levels and white blood cell, hemoglobin, and platelet counts in the individuals examined in our present study.

### Comparison of mRNA expression levels of cytokines in patients and controls

**Figure 1:**
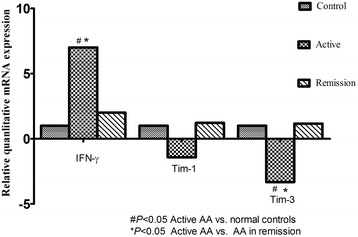
**Relative mRNA expressions of Tim-1**、**Tim-3 and other transcription factors.** Freshly isolated human peripheral blood mononuclear cells (PBMC) from AA patients and healthy controls were quantified by RT-PCR. #*P* < 0.05, AA (newly-diagnosed) vs. normal controls, *P < 0.05, AA (newly-diagnosed) vs. AA(remission).

The data are presented as the fold change in gene expression normalized to an endogenous reference gene and relative to healthy controls and were analyzed using the REST software. IFN-γ was upregulated in active AA patients by 7.0-fold (*P* < 0.05), compared with controls, and by 2.2-fold compared with remission patients (*P* > 0.05). No significant difference in IFN-γ expression was found between SAA patients and the MAA group (*P* > 0.05). For all parameters, there was no significant difference between patients in remission and the control group (Figure 1).

### Tim-1 and Tim-3 expressions in patients and controls

The relative mRNA expression level of Tim-1 was decreased 1.4-fold in active patients, compared with healthy controls (*P* > 0.05), and 1.2-fold, compared with remission patients (*P* > 0.05). The decrease observed in Tim-3 expression was 3.3-fold (*P* < 0.05) and 1.1-fold (*P* > 0.05), in active patients compared with controls and AA patients in remission, respectively. The expression of Tim-1 in SAA patients was found to be unchanged compared with the MAA group. Tim-3 in SAA group displayed a trend towards a decline, but the difference did not reach statistical significance when compared with that of the MAA group.

### The ratio of Tim-3/Tim-1 in normal control individuals and patients with active disease

**Figure 2:**
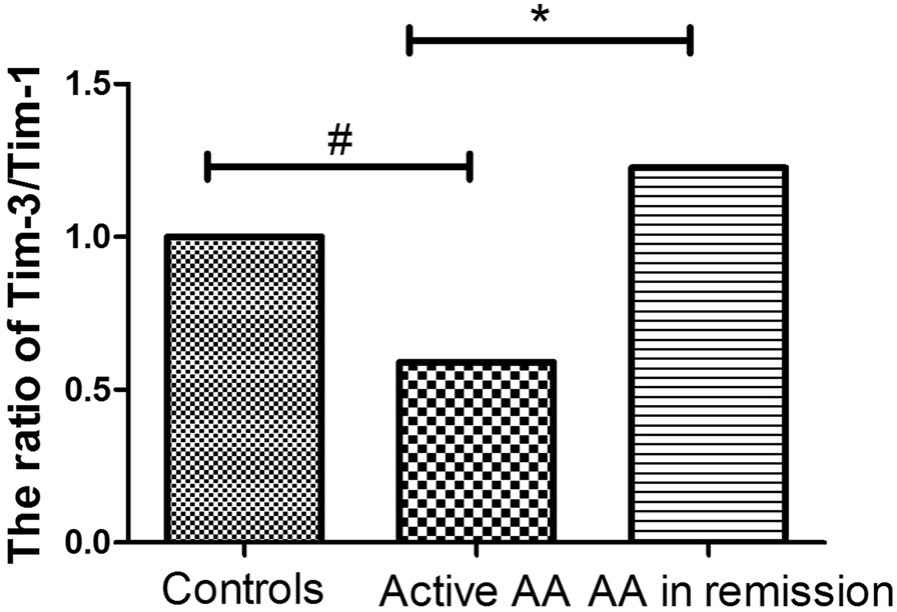
**The Tim-3/Tim-1 ratio in AA patients of newly-diagnosed and AA patients in remission.** The ratio of Tim-3/Tim-1 in active AA patients was decreased significantly when compared with that of healthy controls(#*P* < 0.05) and patients in remission(*P < 0.05).

The ratio of Tim-3/Tim-1 in patients with active disease was decreased significantly when compared with that in individuals in the normal group (*P* < 0.05) and AA patients in remission (*P* < 0.05) (Figure 2). The ratio of Tim-3/Tim-1 in the AA remission group had a trend towards elevation, but this difference did not reach statistical significance when compared with that of control group. Interestingly, the ratio of Tim-3/Tim-1 was found to be unchanged in SAA patients compared to MAA ones. Statistically significant differences were not found in patients in remission, compared to healthy controls (*P* > 0.05).

## Discussion

Aplastic anemia (AA) is a rare, potentially life-threatening failure of hematopoiesis that is characterized by pancytopenia and bone marrow aplasia. It was originally thought to result from a direct toxic effect on hematopoietic stem cells, leading to a decrease in their numbers. An increase in monocyte-derived DCs (DC1) shifted from the stable form to the active one, promoting Th0 cells to polarize into Th1 cells. The alterations in Th1 lymphocyte function and cytokines have been intensively investigated as surrogates of AA and as possible candidates of an index for diagnosis and prognosis [17,18]. In the current study, we examined the function of Th-associated transcription factors in regulating Tim-1 and Tim-3 expression. Patients with active AA disease demonstrated decreased mRNA expression levels of Tim-3, while the levels of Tim-1 in patients were comparable to the levels of healthy controls, thereby suggesting that the balance of Tim-3 and Tim-1 may play an important role in AA pathogenesis.

Tim-3 is a cell surface protein that is expressed at the late stages of IFN-γ-secreting CD4+ Th1 cell differentiation, and it is constitutively expressed in dendritic cells. Altered expression levels of Tim-3, which often correlates with disease activity, have been reported in various experimental and human autoimmune disorders. The administration of a Tim-3-blocking antibody inhibited the suppression activity of Tregs, thereby resulting in augmented production of IFN-γ [19]. Moreover, Ju recently reported that following Tim-3 knockdown using specific shRNAs, a significant increase in IFN-γ production from hepatic CD8+ T cells was observed in a HBV mouse model [20]. Our previous study confirmed that the protein levels of Tim-3 in active AA patients were significantly decreased, compared with normal controls [21]. The observed reduction in Tim-3 protein and mRNA levels in the CD4+ T cells of SAA patients was more obvious than in MAA patients, thereby implying that reduced Tim-3 expression on CD4+ T cells may lead to more intensive CD4+ T cell accumulation. Therefore, up-regulating the expression of Tim-3 in AA patients may also represent a therapeutic approach against AA.

Recently, a new gene encoding human hepatitis A virus cellular receptor (HAVCR), which was also identified as T-cell immunoglobulin-and mucin-domain-containing molecule-1 (Tim-1), has attracted much attention in the field of autoimmune diseases. Tim-1 is a type I transmembrane protein, with extracellular immunoglobulin and mucin domains and intracellular domains of various lengths. Tim-1 was first identified in the African green monkey, and later in humans, as HAVCR, the receptor exploited by hepatitis A for viral entry. It was reported that Tim-1 mRNA is expressed by Th2 cells, but not Th1 cells, in humans and mice. Furthermore, Tim-1 ligation enhanced the proliferation of T cells and, in Th2 cells, the production of IL-4. In vivo administration of anti-Tim-1 antibodies increased production of both IL-4 and IFN-γ in unpolarized cells [9]. In our present study, AA patients had increased IFN-γ and an unchanged Tim-1 mRNA level compared to controls. Tim-1 expression patterns may be related to differences in Th1/Th2 profiles. Umetsu et al. showed that Tim-1 expression is highly increased on newly activated cells. However, with time, Th1 cells lose Tim-1 expression, while it is sustained on Th2 cells [9,11]. Thus, the trend of down-regulated expression of Tim-1 in AA patients is in agreement with the exaggerated Th1 response in AA patients. Although the alteration of Tim-1 or Tim-3 mRNA expression is evident in patients with various autoimmune diseases, such as systemic lupus erythematosus [22], asthma and other autoimmune diseases [23], little information is available regarding the interactions of these transcription factors in AA.

Our results confirmed a reciprocal pattern in mRNA expression of Tim-1 and Tim-3 in AA. Specifically, the mRNA levels of Tim-3 were significantly decreased in active AA patients, compared to controls and patients in remission, while Tim-1 levels were not significantly changed in AA patients. The ratio of Tim-3/Tim-1 in patients with active disease was significantly reduced when compared with that of the normal groups and AA patients in remission. Regulating the balance of Tim-1 and Tim-3 in AA patients may also be a therapeutic approach against AA, although further studies are warranted to validate this concept.
